# Crystal structure, Hirshfeld surface analysis and DFT calculations of (*E*)-3-[1-(2-hy­droxy­phenyl­anilino)ethyl­idene]-6-methyl­pyran-2,4-dione

**DOI:** 10.1107/S2056989022007514

**Published:** 2022-07-29

**Authors:** Imane Faraj, Ali Oubella, Karim Chkirate, Khalil Al Mamari, Tuncer Hökelek, Joel T. Mague, Lhoussaine El Ghayati, Nada Kheira Sebbar, El Mokhtar Essassi

**Affiliations:** a Laboratory of Heterocyclic Organic Chemistry, Medicines Science Research Center, Pharmacochemistry Competence Center, Mohammed V University in Rabat, Faculté des Sciences, Av. Ibn Battouta, BP 1014, Rabat, Morocco; b Laboratory of Chemistry and Environment, Applied Bioorganic Chemistry Team, Faculty of Sciences, Ibn Zohr University, Agadir, Morocco; c Laboratoire de Synthése Organique et Physico-Chimie Moléculaire, Département de Chimie, Faculté des Sciences, Semlalia, B.P 2390, Marrakech 40001, Morocco; d Department Of Chemistry – Faculty of Education – University of Hodiedah, Yemen; e Department of Physics, Hacettepe University, 06800 Beytepe, Ankara, Turkey; f Department of Chemistry, Tulane University, New Orleans, LA 70118, USA; Venezuelan Institute of Scientific Research, Venezuela

**Keywords:** crystal structure, hydrogen bond, pyran­one, phenol

## Abstract

The asymmetric unit of the title compound, C_14_H_13_NO_4_, contains three independent mol­ecules, which differ slightly in conformation. Each contains an intra­molecular N—H⋯O hydrogen bond. In the crystal, O—H⋯O hydrogen bonds form chains of mol­ecules, which are linked into corrugated sheets parallel to (  03) plane by C—H⋯O hydrogen bonds together with π inter­actions between the carbonyl groups and the 2-hy­droxy­phenyl rings. The layers are linked by further C—H⋯O hydrogen bonds. The Hirshfeld surface analysis of the crystal structure indicates that the most important contributions for the crystal packing are from H⋯H (49.0%), H⋯O/O⋯H (28.3%) and H⋯C/C⋯H (10.9%) inter­actions. van der Waals inter­actions are the dominant inter­actions in the crystal packing. Moreover, density functional theory (DFT) optimized structures at the B3LYP/ 6–311 G(d,p) level are compared with the experimentally determined mol­ecular structure in the solid state. The HOMO–LUMO behavior was elucidated to determine the energy gap of 4.53 eV.

## Abstract 2

The asymmetric unit contains three independent mol­ecules differing slightly in conformation. Portions of the observed conformations are determined by intra­molecular N—H⋯O hydrogen bonds. In the crystal, O—H⋯O hydrogen bonds form chains of mol­ecules which are linked into corrugated sheets parallel to ( 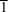 03) by C—H⋯O hydrogen bonds together with π inter­actions between the carbonyl groups and 2-hy­droxy­phenyl rings. The layers are linked by further C—H⋯O hydrogen bonds.

## 1. Chemical context

**Figure 1:**
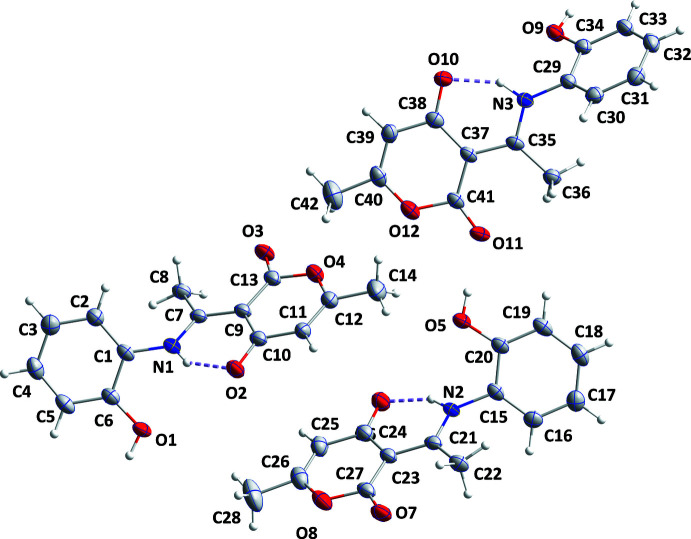
The asymmetric unit with the atom-labeling scheme and 50% probability ellipsoids. The intra­molecular hydrogen bonds are depicted by dashed lines.

Heterocyclic mol­ecules play a very important role in life processes and are of major inter­est in the industrial development of dyes, pharmaceuticals, pesticides, and natural products (Saber *et al.*, 2020 ▸; El Ghayati *et al.*, 2021 ▸; Patra & Saxena, 2010 ▸). Therefore, scientists have devoted considerable effort to finding efficient synthetic methods for a wide variety of heterocyclic compounds (Yeh *et al.*, 2014 ▸; Liaw *et al.*, 2015 ▸). Among these mol­ecules, pyrone derivatives constitute an important class in the heterocycle family since the pyrone structural unit is found in a wide variety of natural bioactive compounds (McGlacken & Fairlamb, 2005 ▸; Beckert *et al.*, 1997 ▸) and also in a wide range of synthetic products with demonstrated efficacy in various fields such as the pharmaceutical and therapeutic field as cytotoxic (Calderón-Montaño *et al.*, 2013 ▸), anti­tumor (Suzuki *et al.*, 1997 ▸; Kondoh *et al.*, 1998 ▸) and anti­microbial agents (Fairlamb *et al.*, 2004 ▸). Another representative example of the pyrone class of compounds, kavalactones, possess many biological activities such as anti­tuberculosis, local anesthetic, anti­convulsant, analgesic, anti­malarial, and sedative activities (Altomare *et al.*, 1997 ▸; Scherer, 1998 ▸; Bilia *et al.*, 2002 ▸; Ernst, 2007 ▸). In this work, we report the synthesis of (*E*)-3-[1-(2-hy­droxy­phenyl­anilino)ethyl­idene]-6-methyl­pyran-2,4-dione, (I) (Fig. 1 ▸) in good yield by the condensation of 2-amino­phenol and dehydroacetic acid along with its crystal and mol­ecular structures as well as the Hirshfeld surface analysis and the density functional theory (DFT) computational calculations carried out at the B3LYP/6–311G(d,p) levels. 
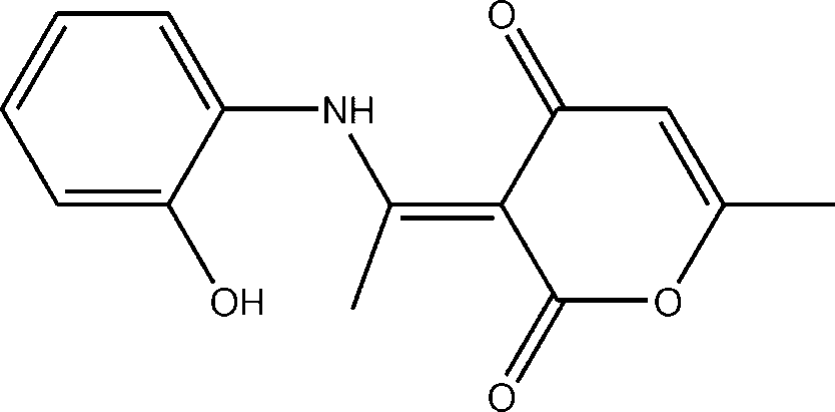


## 2. Structural commentary

The asymmetric unit of the title compound comprises three independent mol­ecules, two of which (those containing O5 and O9) differ modestly in the orientations of the methyl groups while the third differs more in conformation from the other two (Fig. 1 ▸). In each mol­ecule, the conformation is partially determined by an intra­molecular N—H⋯O hydrogen bond (Fig. 1 ▸ and Table 1 ▸), which can be described as a resonance-assisted hydrogen bond (RAHB). With reference to the scheme below, in the three independent mol­ecules the bonds designated **a** are the same within experimental error. The same is true for each of the bonds labeled **b**–**f** and the average values are **a** = 1.323 (3) Å, **b** = 1.431 (3) Å, **c** = 1.447 (3) Å, **d** = 1.433 (3) Å, **e** = 1.226 (3) Å and **f** = 1.254 (3) Å. These compare quite favorably with those found in mol­ecules with *R* = Me (Gilli *et al.*, 2000 ▸) and 4-*X*C_6_H_4_ (*X* = F, Cl, Br; Boulemche *et al.*, 2019 ▸) and accompanied by in depth discussions of the RAHB. 
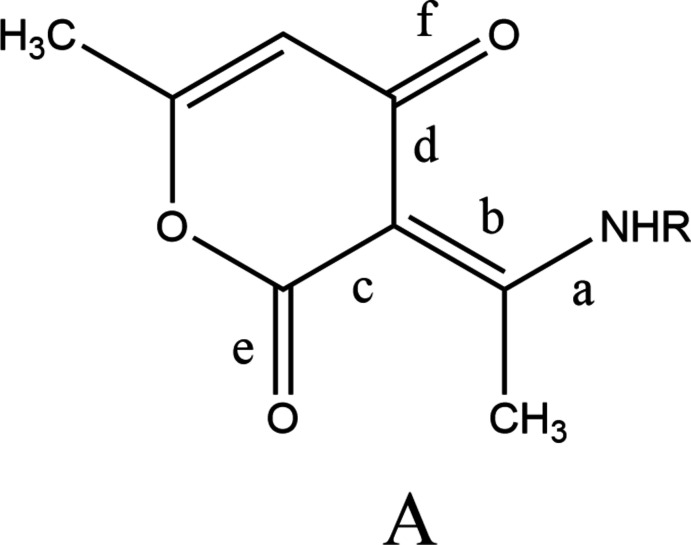


## 3. Supra­molecular features

**Figure 2:**
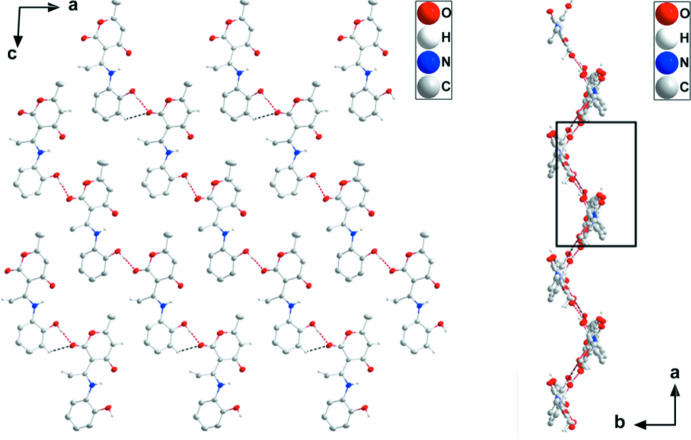
A portion of one layer viewed along the *b*-axis direction (left) and along the *c*-axis direction (right) with O—H⋯O and C—H⋯O hydrogen bonds depicted, respectively, by red and black dashed lines. Non-inter­acting H atoms are omitted for clarity.

**Figure 3:**
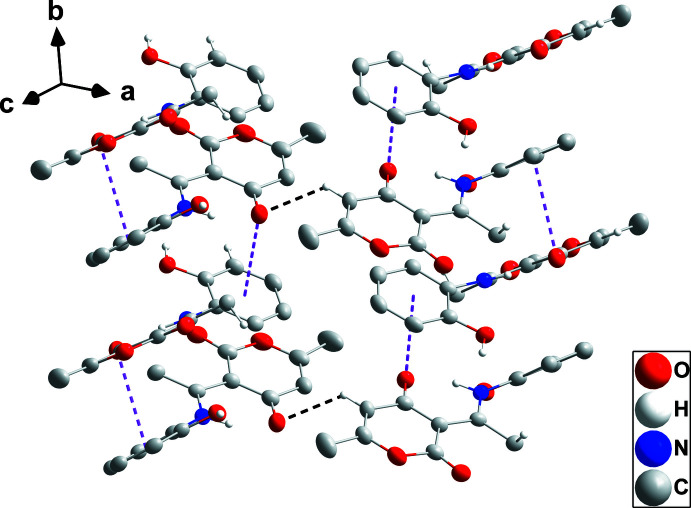
Detail of the C=O⋯π(ring) stacking inter­actions (pink dashed lines) and the connection of stacks by C—H⋯O hydrogen bonding (black dashed lines). Non-inter­acting H atoms are omitted for clarity.

In the crystal, chains containing all three independent mol­ecules are formed by O1—H1*B*⋯O7, O5—H5*B*⋯O11 and O9—H9*B*⋯O3 hydrogen bonds repeating in that order (Table 1 ▸ and Fig. 2 ▸). The chains are linked into corrugated layers parallel to the ( 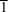 03) plane by C8—H8*C*⋯O8, C33—H33⋯O3 and C36—H36*B*⋯O12 hydrogen bonds together with π inter­actions (Fig. 3 ▸) between the carbonyl groups and the 2-hy­droxy­phenyl rings [O2⋯*Cg*2 = 3.4827 (18) Å, C10⋯*Cg*2 = 3.731 (2) Å, C10=O2⋯*Cg*2 = 91.41 (13)° (*Cg*2 is the centroid of the C1–C6 ring at −x + 3/2, *y* + 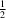 , −*z* + 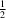 ); O6⋯*Cg*6 = 3.451 (2) Å, C24⋯*Cg*6 = 3.694 (2) Å, C24=O6⋯*Cg*6 = 91.12 (14)° (*Cg*6 is the centroid of the C29-C34 ring at *x*, *y*, *z*); O10⋯*Cg*4 = 3.4110 (18) Å, C38⋯*Cg*4 = 3.656 (2) Å, C38=O10⋯*Cg*4 = 91.00 (13)° (*Cg*4 is the centroid of the C15⋯C20 ring at *x*, *y* − 1, *z*)]. The layers are held together by C11—H11*C*⋯O6 hydrogen bonds (Table 1 ▸ and Fig. 3 ▸).

## 4. Hirshfeld surface analysis

**Figure 4:**
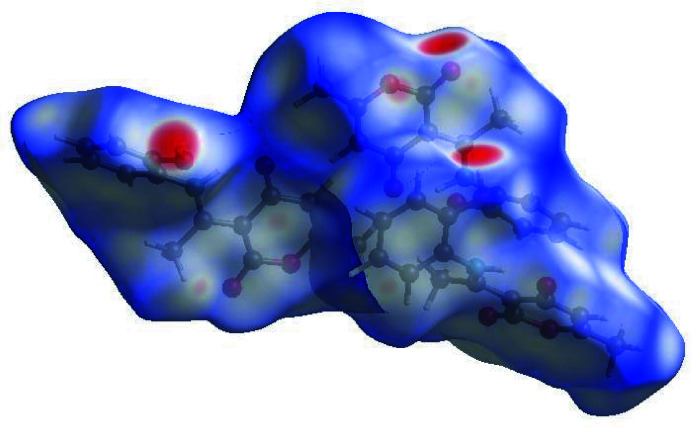
View of the three-dimensional Hirshfeld surface of the title compound, plotted over *d*_norm_ in the range −0.7208 to 1.5611 a.u.

**Figure 5:**
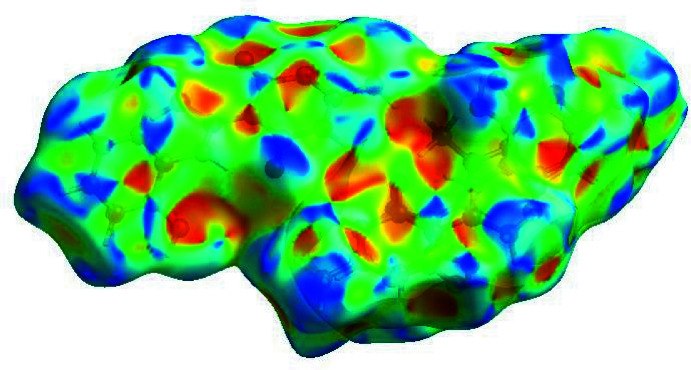
Hirshfeld surface of the title compound plotted over shape-index.

**Figure 6:**
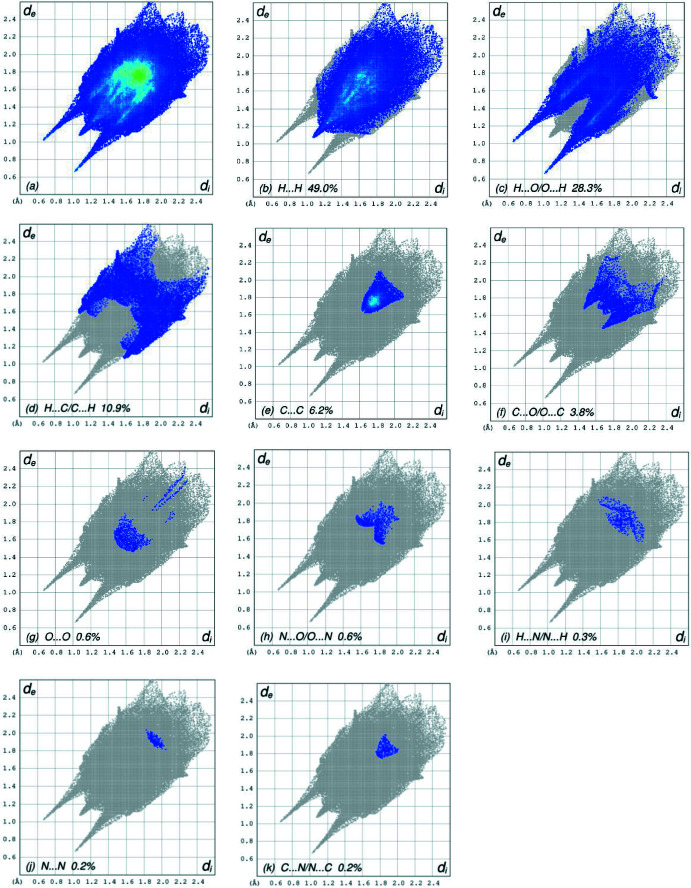
The full two-dimensional fingerprint plots for the title compound, showing (*a*) all inter­actions, and delineated into (*b*) H⋯H, (*c*) H⋯O/O⋯H, (*d*) H⋯C/C⋯H, (*e*) C⋯C, (*f*) C⋯O/O⋯C, (*g*) O⋯O, (*h*) N⋯O/O⋯N, (*i*) H⋯N/N⋯H, (*j*) N⋯N and (*k*) C⋯N/N⋯C inter­actions. The *d*_i_ and *d*_e_ values are the closest inter­nal and external distances (in Å) from given points on the Hirshfeld surface.

In order to visualize the inter­molecular inter­actions, a Hirshfeld surface (HS) analysis (Hirshfeld, 1977 ▸) was carried out using *Crystal Explorer 17.5* (Turner *et al.*, 2017 ▸). In the HS plotted over *d*_norm_ (Fig. 4 ▸), the white surface indicates contacts with distances equal to the sum of van der Waals radii, and the red and blue colors indicate distances shorter (in close contact) or longer (distinct contact) than the sum of the van der Waals radii, respectively (Venkatesan *et al.*, 2016 ▸). The shape-index of the HS is a tool to visualize π–π stacking by the presence of adjacent red and blue triangles; if there are no adjacent red and/or blue triangles, then there are no π–π inter­actions. Fig. 5 ▸ clearly suggests that there are π–π inter­actions in (I). The overall two-dimensional fingerprint plot, Fig. 6 ▸ *a*, and those delineated into H⋯H, H⋯O/O⋯H, H⋯C/C⋯H, C⋯C, C⋯O/O⋯C, O⋯O, N⋯O/O⋯N, H⋯N/N⋯H, N⋯N and C⋯N/N⋯C contacts (McKinnon *et al.*, 2007 ▸) are illustrated in Fig. 6 ▸ *b*–*k*, respectively, together with their relative contributions to the Hirshfeld surface. The most important inter­action is H⋯H contributing 49.0% to the overall crystal packing, which is reflected in Fig. 6 ▸ *b* as widely scattered points of high density due to the large hydrogen content of the mol­ecule with the tip at *d*_e_ = *d*_i_ = 1.09 Å. The pair of spikes in in the fingerprint plot delineated into H⋯O/O⋯H contacts with a 28.3% contribution to the HS, Fig. 6 ▸ *c*, has a symmetric distribution of points with the tips at *d*_e_ + *d*_i_ = 1.69 Å. In the presence of C—H⋯π inter­actions, the pair of characteristic wings in the fingerprint plot delineated into H⋯C/C⋯H contacts, Fig. 6 ▸ *d*, with a 10.9% contribution to the HS has the tips at *d*_e_ + *d*_i_ = 2.67 Å. The C⋯C contacts, Fig. 6 ▸ *e*, with a 6.2% contribution to the HS have a bullet-shaped distribution of points and the tip at *d*_e_ = *d*_i_ = 1.64 Å. The symmetric distrib­ution of points for the C⋯O/O⋯C contacts, Fig. 6 ▸ *f*, with 3.8% contribution to the HS has a pair of the scattered points of spikes with the tips at *d*_e_ + *d*_i_ = 3.11 Å. Finally, the contributions of the remaining O⋯O, N⋯O/O⋯N, H⋯N/N⋯H, N⋯N and C⋯N/N⋯C contacts (Fig. 6 ▸ *g*–*k*) are smaller than 1.0% with low densities of points.

**Figure 7:**
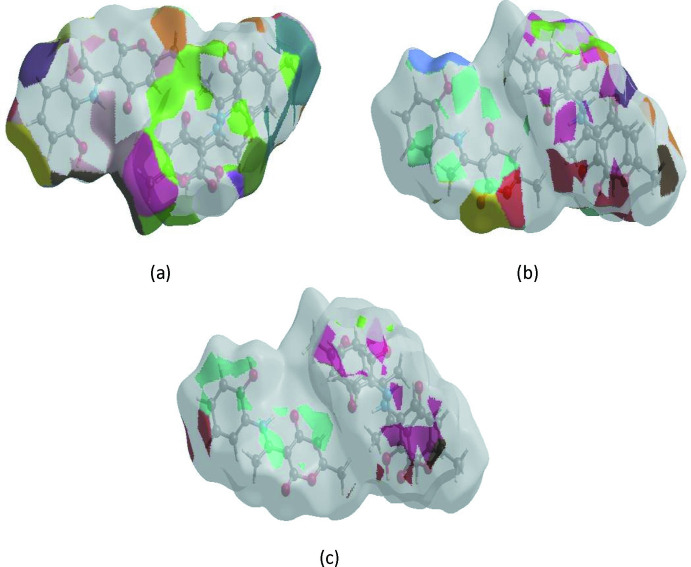
The Hirshfeld surface representations with the function *d*_norm_ plotted onto the surface for (*a*) H⋯H, (*b*) H⋯O/O⋯H and (*c*) H⋯C/C⋯H inter­actions.

The Hirshfeld surface representations with the function *d*_norm_ plotted onto the surface are shown for the H⋯H, H⋯O/O⋯H and H⋯C/C⋯H inter­actions in Fig. 7 ▸ *a*–*c*, respectively. The Hirshfeld surface analysis confirms the importance of H-atom contacts in establishing the packing. The large number of H⋯H, H⋯O/O⋯H and H⋯C/C⋯H inter­actions suggest that van der Waals inter­actions play the major role in the crystal packing (Hathwar *et al.*, 2015 ▸).

## 5. DFT calculations

**Figure 8:**
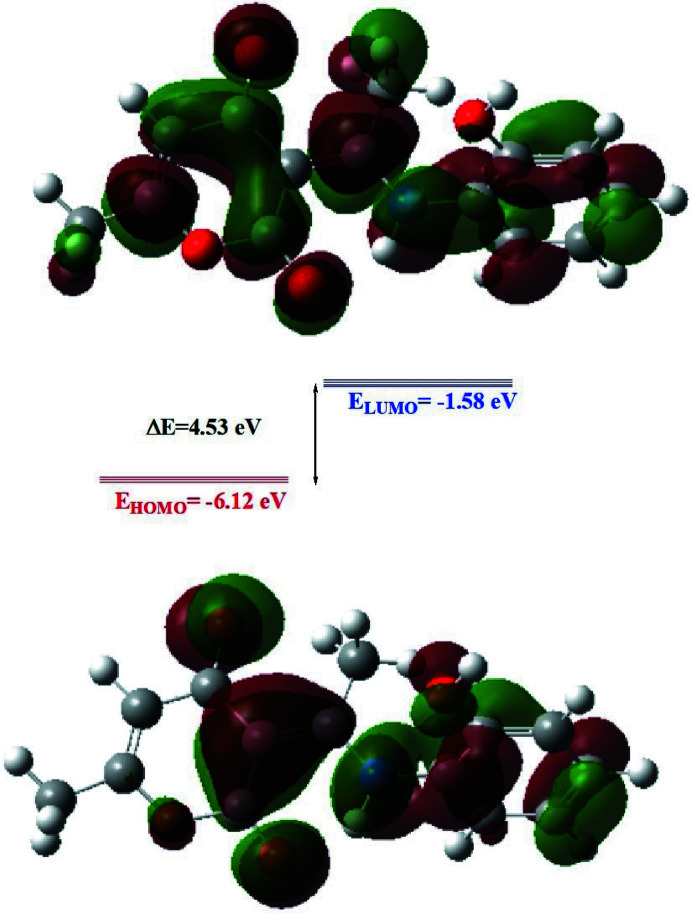
The energy band gap of the title compound.

The optimized structure of the title compound in the gas phase was generated theoretically *via* density functional theory (DFT) using the standard B3LYP functional and 6–311 G(d,p) basis-set calculations (Becke, 1993 ▸) as implemented in *GAUSSIAN 09* (Frisch *et al.*, 2009 ▸). The theoretical and experimental results are in good agreement (Table 2 ▸). The highest-occupied mol­ecular orbital (HOMO), acting as an electron donor, and the lowest-unoccupied mol­ecular orbital (LUMO), acting as an electron acceptor, are very important parameters for quantum chemistry. When the energy gap is small, the mol­ecule is highly polarizable and has high chemical reactivity. The DFT calculations provide some important information on the reactivity and site selectivity of the mol­ecular framework. *E*_HOMO_ and *E*_LUMO_, which clarify the inevitable charge-exchange collaboration inside the mol­ecule, electronegativity (χ), hardness (η), potential (μ), electrophilicity (ω) and softness (σ) are recorded in Table 3 ▸. The significance of η and σ is to evaluate both the reactivity and stability. The electron transition from the HOMO to the LUMO energy level is shown in Fig. 8 ▸. The HOMO and LUMO are localized in the plane extending from the whole (*E*)-3-[1-(2-hy­droxy­phenyl­amino)­ethyl­idene]-6-methyl-3*H*-pyran-2,4-dione ring. The energy band gap [Δ*E* = *E*_LUMO_ − *E*_HOMO_] of the mol­ecule is 4.54 eV, and the frontier mol­ecular orbital energies, *E*_HOMO_ and *E*_LUMO_ are −6.12 and −1.58 eV, respectively.

## 6. Mol­ecular electrostatic (MEP)

**Figure 9:**
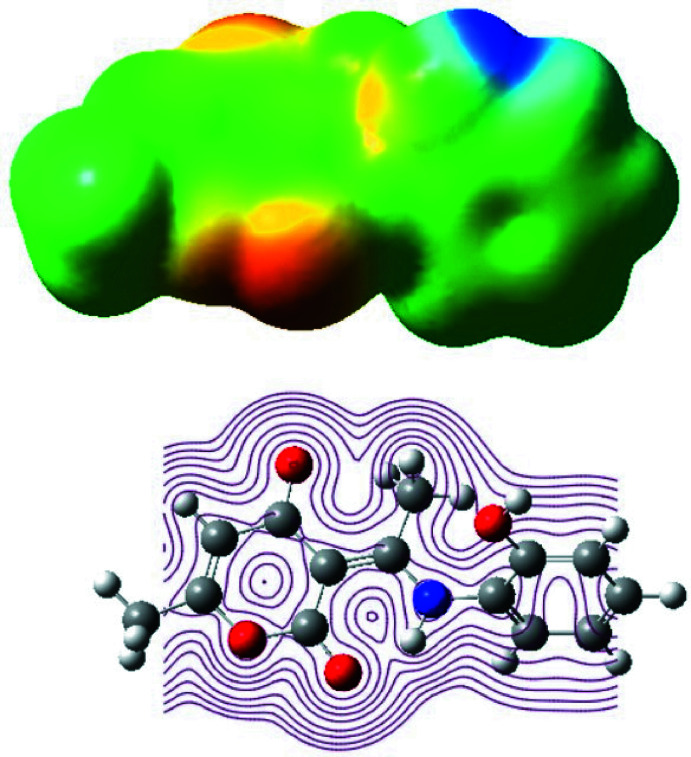
MEP surfaces mapped from the optimized geometries of the B3LYP/6–311 G calculation.

Mol­ecular electrostatic potential (MEP) was used to broadly predict reactive sites for electrophilic and nucleophilic attack in the title compound by B3LYP/6-31G optimized geometries using *Gaussview* software (Frisch *et al.*, 2009 ▸). The total electron density onto which the electrostatic potential surface has been mapped is shown in Fig. 9 ▸. This figure gives a visual representation of the chemically active sites and comparative reactivity of atoms where red regions denote the most negative electrostatic potential, blue represents regions of the most positive electrostatic potential, and green represents the region of zero potential. The distribution favors the existence of the intra and inter­molecular C—H⋯O and N—H⋯O hydrogen bonding.

## 7. Database survey

A search of the Cambridge Structural Database (CSD, version 5.43, updated to March 2022; Groom *et al.*, 2016 ▸) for the fragment *A* (allowing *R* to be any substituent) yielded 66 hits of which 15 were deemed most similar to the title mol­ecule. These include mol­ecules with *R* = Me (FOTQOW; Kwocz *et al.*, 2015 ▸), *p*-anis (GOWYOG, Gilli *et al.*, 2000 ▸), 4-ClC_6_H_4_ (GOXLOU, GOXLOU02; Boulemche *et al.*, 2019 ▸), 4-BrC_6_H_4_ (VOPLOC01; Boulemche *et al.*, 2019 ▸), Et (HABNED; Xiao *et al.*, 1993 ▸), H (HIVTUD; Seijas *et al.* 2014 ▸), Ph (PAEXPY; Gilli *et al.*, 2000 ▸), 4-H_2_NC_6_H_4_ (QADRIY; Užarević *et al.* 2010 ▸), 4-EtOC_6_H_4_ (QEQQEL; Djedouani *et al.*, 2018 ▸), 4-MeOC_6_H_4_CH_2_ (XECGEV; Wang *et al.*, 2022 ▸), PhCH(Me) (XECGOF; Wang *et al.*, 2022 ▸) and 2-CH_2_C_5_H_4_N (XECHEW; Wang *et al.*, 2022 ▸). Although not all of these reports discuss the intra­molecular N—H⋯O hydrogen bonds in detail, it is clear that all have very similar metrical parameters to one another and to those in the title mol­ecule.

## 8. Synthesis and crystallization

To a solution of 2-amino­phenol (2.5 mmol) in 30 mL of ethanol, 2.5 mmol of de­hydro­acetic acid were added. The mixture was refluxed for 1 h. After cooling, the precipitate that formed was recrystallized from ethanol solution to give yellow crystals in 88% yield.

## 9. Refinement

Crystal, data collection and refinement details are presented in Table 4 ▸. Hydrogen atoms were included as riding contributions in idealized positions (O—H = 0.87 Å, N—H = 0.91 Å, C—H = 0.95–0.98 Å) with *U*_iso_(H) = 1.2*U*_eq_(C, N) or 1.5*U*_eq_(O, C-meth­yl).

## Supplementary Material

Crystal structure: contains datablock(s) I, global. DOI: 10.1107/S2056989022007514/zn2020sup1.cif

Structure factors: contains datablock(s) I. DOI: 10.1107/S2056989022007514/zn2020Isup2.hkl

Supporting information file. DOI: 10.1107/S2056989022007514/zn2020Isup3.cml

CCDC reference: 2192044

Additional supporting information:  crystallographic information; 3D view; checkCIF report

## Figures and Tables

**Table 1:** Hydrogen-bond geometry (Å, °)

*D*—H⋯*A*	*D*—H	H⋯*A*	*D*⋯*A*	*D*—H⋯*A*
O1—H1*B*⋯O7^i^	0.87	1.79	2.662 (2)	177
N1—H1*A*⋯O2	0.91	1.72	2.538 (3)	148
C8—H8*C*⋯O8^ii^	0.98	2.48	3.441 (3)	167
C11—H11⋯O6	0.95	2.57	3.253 (3)	129
O5—H5*B*⋯O11^iii^	0.87	1.83	2.689 (2)	170
N2—H2*A*⋯O6	0.91	1.71	2.539 (3)	151
O9—H9*B*⋯O3^iv^	0.87	1.82	2.691 (2)	179
N3—H3*A*⋯O10	0.91	1.71	2.532 (3)	148
C33—H33⋯O3^iv^	0.95	2.53	3.225 (3)	130
C36—H36*B*⋯O12^v^	0.98	2.56	3.531 (3)	173

Symmetry codes: (i) 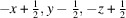 ; (ii) 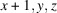 ; (iii) 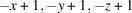 ; (iv) 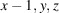 ; (v) 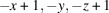 .

**Table 2:** Comparison of selected (X-ray and DFT) geometric data (Å, °)

Bonds/angles	X-ray	B3LYP/6–311G(d,p)
O1—C6	1.361 (3)	1.38765
O2—C10	1.253 (3)	1.255
O3—C13	1.228 (3)	1.265
O4—C12	1.381 (3)	1.395
N1—C7	1.326 (3)	1.349
O4—C13	1.389 (3)	1.409
N1—C1	1.420 (3)	1.427
C1—C2	1.392 (3)	1.401
C1—C6	1.397 (3)	1.399
C2—C3	1.373 (4)	1.388
C3—C4	1.393 (4)	1.399
C4—C5	1.384 (4)	1.398
C5—C6	1.390 (3)	1.399
C9—C13	1.435 (3)	1.445
		
C12—O4—C13	121.9 (2)	122.02
C7—N1—C1	126.7 (2)	127.03
C7—N1—H1*A*	111.6	114.24
C1—N1—H1*A*	121.7	122.06
C2—C1—C6	120.4 (2)	120.94
C2—C1—N1	121.4 (2)	121.36
C6—C1—N1	118.1 (2)	119.02
C3—C2—C1	120.1 (2)	120.60
C5—C4—C3	120.8 (2)	120.14
C4—C5—C6	119.8 (2)	120.18
N1—C7—C9	117.5 (2)	119.48
N1—C7—C8	119.1 (2)	122.41
O4—C12—C14	112.5 (2)	112.80
O3—C13—O4	114.2 (2)	114.26

**Table 3:** Calculated energies.

Mol­ecular energy	Compound (I)
Total energy, TE (eV)	−24399.73
E_HOMO_ (eV)	−6.12
E_LUMO_ (eV)	−1.58
Gap, ΔE/i> (eV)	4.53
Dipole moment, μ (Debye)	4.1895
Ionization potential, I (eV)	6.12
Electron affinity, A	1.58
Electronegativity, χ	3.85
Hardness, η	2.27
Electrophilicity index, ω	3.27
Softness, σ	0.44
Fraction of electron transferred, ΔN	0.69

**Table 4:** Experimental details

Crystal data	
Chemical formula	C_14_H_13_NO_4_
*M*_r_	259.25
Crystal system, space group	Monoclinic, *P*2_1_/*n*
Temperature (K)	150
*a*, *b*, *c* (Å)	11.6407 (4), 7.4412 (2), 42.2828 (12)
β (°)	93.038 (2)
*V* (Å^3^)	3657.42 (19)
*Z*	12
Radiation type	Cu *K*α
μ (mm^−1^)	0.87
Crystal size (mm)	0.27 × 0.07 × 0.07

Data collection	
Diffractometer	Bruker D8 VENTURE PHOTON 100 CMOS
Absorption correction	Multi-scan (*SADABS*; Krause *et al.*, 2015 ▸)
*T*_min_, *T*_max_	0.82, 0.94
No. of measured, independent and observed [*I* > 2σ(*I*)] reflections	30476, 7135, 5024
*R*_int_	0.070
(sin θ/λ)_max_ (Å^−1^)	0.618

Refinement	
*R*[*F* ^2^ > 2σ(*F* ^2^)], *wR*(*F* ^2^), *S*	0.056, 0.146, 1.04
No. of reflections	7135
No. of parameters	520
H-atom treatment	H-atom parameters constrained
Δρ_max_, Δρ_min_ (e Å^−3^)	0.49, −0.43

Computer programs: *APEX3* and *SAINT* (Bruker, 2016 ▸), *SHELXT5* (Sheldrick, 2015*a* ▸), *SHELXL2018/3* (Sheldrick, 2015*b* ▸), *DIAMOND* (Brandenburg & Putz, 2012 ▸) and *SHELXTL* (Sheldrick, 2008 ▸).

## supplementary crystallographic information

### Crystal data

**Table:**

C_14_H_13_NO_4_	*F*(000) = 1632
*M**_r_* = 259.25	*D*_x_ = 1.412 Mg m^−^^3^
Monoclinic, *P*2_1_/*n*	Cu *K*α radiation, λ = 1.54178 Å
*a* = 11.6407 (4) Å	Cell parameters from 9858 reflections
*b* = 7.4412 (2) Å	θ = 4.0–72.3°
*c* = 42.2828 (12) Å	µ = 0.87 mm^−^^1^
β = 93.038 (2)°	*T* = 150 K
*V* = 3657.42 (19) Å^3^	Plate, colourless
*Z* = 12	0.27 × 0.07 × 0.07 mm

### Data collection

**Table:**

Bruker D8 VENTURE PHOTON 100 CMOS diffractometer	7135 independent reflections
Radiation source: INCOATEC IµS micro–focus source	5024 reflections with *I* > 2σ(*I*)
Mirror monochromator	*R*_int_ = 0.070
Detector resolution: 10.4167 pixels mm^-1^	θ_max_ = 72.4°, θ_min_ = 3.9°
ω scans	*h* = −13→14
Absorption correction: multi-scan (*SADABS*; Krause *et al.*, 2015)	*k* = −8→9
*T*_min_ = 0.82, *T*_max_ = 0.94	*l* = −52→51
30476 measured reflections	

### Refinement

**Table:**

Refinement on *F*^2^	Primary atom site location: dual
Least-squares matrix: full	Secondary atom site location: difference Fourier map
*R*[*F*^2^ > 2σ(*F*^2^)] = 0.056	Hydrogen site location: mixed
*wR*(*F*^2^) = 0.146	H-atom parameters constrained
*S* = 1.04	*w* = 1/[σ^2^(*F*_o_^2^) + (0.0569*P*)^2^ + 2.375*P*] where *P* = (*F*_o_^2^ + 2*F*_c_^2^)/3
7135 reflections	(Δ/σ)_max_ < 0.001
520 parameters	Δρ_max_ = 0.49 e Å^−^^3^
0 restraints	Δρ_min_ = −0.43 e Å^−^^3^

### Special details

**Table:**

Geometry. All esds (except the esd in the dihedral angle between two l.s. planes) are estimated using the full covariance matrix. The cell esds are taken into account individually in the estimation of esds in distances, angles and torsion angles; correlations between esds in cell parameters are only used when they are defined by crystal symmetry. An approximate (isotropic) treatment of cell esds is used for estimating esds involving l.s. planes.
Refinement. Refinement of F^2^ against ALL reflections. The weighted R-factor wR and goodness of fit S are based on F^2^, conventional R-factors R are based on F, with F set to zero for negative F^2^. The threshold expression of F^2^ > 2sigma(F^2^) is used only for calculating R-factors(gt) etc. and is not relevant to the choice of reflections for refinement. R-factors based on F^2^ are statistically about twice as large as those based on F, and R- factors based on ALL data will be even larger. H-atoms attached to carbon were placed in calculated positions (C—H = 0.95 - 0.98 Å) while those attached to nitrogen and to oxygen were placed in locations derived from a difference map and their parameters adjusted to give N—H = 0.91 and O—H = 0.87 Å. All were included as riding contributions with isotropic displacementparameters 1.2 - 1.5 times those of the attached atoms.

### Fractional atomic coordinates and isotropic or equivalent isotropic displacement parameters (Å^2^)

**Table:**

	*x*	*y*	*z*	*U*_iso_*/*U*_eq_	
O1	0.61999 (14)	0.3312 (2)	0.21067 (4)	0.0333 (4)	
H1B	0.582256	0.290388	0.193862	0.050*	
O2	0.58889 (14)	0.5447 (2)	0.29280 (4)	0.0341 (4)	
O3	0.92249 (14)	0.2912 (2)	0.34495 (4)	0.0357 (4)	
O4	0.77660 (15)	0.3859 (2)	0.37143 (4)	0.0383 (4)	
N1	0.75437 (16)	0.4599 (3)	0.25901 (5)	0.0266 (4)	
H1A	0.682525	0.495459	0.263795	0.032*	
C1	0.7928 (2)	0.4732 (3)	0.22776 (6)	0.0265 (5)	
C2	0.8949 (2)	0.5605 (3)	0.22159 (6)	0.0297 (5)	
H2	0.941384	0.610471	0.238523	0.036*	
C3	0.9286 (2)	0.5746 (3)	0.19105 (6)	0.0352 (6)	
H3	0.998909	0.632341	0.186842	0.042*	
C4	0.8590 (2)	0.5037 (4)	0.16627 (6)	0.0361 (6)	
H4	0.883123	0.511350	0.145199	0.043*	
C5	0.7553 (2)	0.4223 (3)	0.17201 (6)	0.0329 (6)	
H5	0.707586	0.377192	0.154888	0.040*	
C6	0.7211 (2)	0.4068 (3)	0.20290 (6)	0.0279 (5)	
C7	0.81395 (19)	0.3983 (3)	0.28431 (6)	0.0254 (5)	
C8	0.92598 (19)	0.3054 (3)	0.28010 (6)	0.0300 (5)	
H8A	0.935580	0.284869	0.257501	0.045*	
H8B	0.926635	0.189818	0.291232	0.045*	
H8C	0.989161	0.380487	0.288817	0.045*	
C9	0.76450 (19)	0.4190 (3)	0.31430 (6)	0.0269 (5)	
C10	0.6510 (2)	0.4963 (3)	0.31640 (6)	0.0285 (5)	
C11	0.6080 (2)	0.5161 (3)	0.34727 (6)	0.0291 (5)	
H11	0.534842	0.570016	0.349396	0.035*	
C12	0.6685 (2)	0.4605 (3)	0.37321 (6)	0.0334 (6)	
C13	0.8268 (2)	0.3612 (3)	0.34269 (6)	0.0299 (5)	
C14	0.6307 (3)	0.4684 (5)	0.40622 (7)	0.0560 (8)	
H14A	0.551287	0.512407	0.406083	0.084*	
H14B	0.681053	0.550015	0.418746	0.084*	
H14C	0.634724	0.347979	0.415605	0.084*	
O5	0.38904 (14)	0.6512 (2)	0.45296 (4)	0.0318 (4)	
H5B	0.442098	0.689109	0.466620	0.048*	
O6	0.35346 (14)	0.4346 (3)	0.37210 (4)	0.0377 (4)	
O7	−0.00724 (15)	0.6953 (2)	0.34030 (4)	0.0374 (4)	
O8	0.12052 (17)	0.6280 (2)	0.30612 (4)	0.0418 (5)	
N2	0.21857 (16)	0.5135 (3)	0.41527 (5)	0.0271 (4)	
H2A	0.282524	0.476791	0.405602	0.033*	
C15	0.21047 (19)	0.5018 (3)	0.44869 (6)	0.0264 (5)	
C16	0.1203 (2)	0.4113 (3)	0.46191 (6)	0.0298 (5)	
H16	0.061519	0.357723	0.448597	0.036*	
C17	0.1160 (2)	0.3992 (3)	0.49450 (6)	0.0331 (6)	
H17	0.054670	0.337409	0.503716	0.040*	
C18	0.2028 (2)	0.4786 (3)	0.51356 (6)	0.0350 (6)	
H18	0.198635	0.474495	0.535929	0.042*	
C19	0.2955 (2)	0.5638 (3)	0.50054 (6)	0.0318 (5)	
H19	0.355280	0.614281	0.513917	0.038*	
C20	0.30016 (19)	0.5747 (3)	0.46783 (6)	0.0272 (5)	
C21	0.14059 (19)	0.5724 (3)	0.39396 (6)	0.0261 (5)	
C22	0.0313 (2)	0.6523 (3)	0.40471 (6)	0.0321 (5)	
H22A	0.037677	0.669862	0.427702	0.048*	
H22B	−0.032943	0.571023	0.399231	0.048*	
H22C	0.017599	0.768387	0.394222	0.048*	
C23	0.1680 (2)	0.5631 (3)	0.36137 (6)	0.0283 (5)	
C24	0.2774 (2)	0.4919 (3)	0.35259 (6)	0.0320 (5)	
C25	0.3014 (2)	0.4926 (3)	0.31974 (6)	0.0346 (6)	
H25	0.371930	0.444072	0.313268	0.042*	
C26	0.2256 (3)	0.5610 (3)	0.29800 (7)	0.0407 (6)	
C27	0.0887 (2)	0.6317 (3)	0.33735 (6)	0.0321 (5)	
C28	0.2375 (4)	0.5756 (5)	0.26316 (7)	0.0691 (11)	
H28A	0.316636	0.546088	0.258193	0.104*	
H28B	0.219602	0.698620	0.256236	0.104*	
H28C	0.184278	0.491755	0.252138	0.104*	
O9	0.06273 (13)	0.1503 (2)	0.39114 (4)	0.0304 (4)	
H9B	0.017440	0.197100	0.376319	0.046*	
O10	0.10567 (13)	−0.0837 (2)	0.47274 (4)	0.0321 (4)	
O11	0.46131 (14)	0.1935 (2)	0.50481 (4)	0.0326 (4)	
O12	0.33818 (15)	0.1056 (2)	0.53955 (4)	0.0347 (4)	
N3	0.23507 (16)	0.0148 (3)	0.42968 (5)	0.0263 (4)	
H3A	0.171281	−0.027001	0.438829	0.032*	
C29	0.24459 (19)	0.0095 (3)	0.39632 (6)	0.0260 (5)	
C30	0.3378 (2)	−0.0727 (3)	0.38300 (6)	0.0304 (5)	
H30	0.396873	−0.125418	0.396305	0.036*	
C31	0.3448 (2)	−0.0780 (3)	0.35050 (6)	0.0341 (6)	
H31	0.408973	−0.132571	0.341388	0.041*	
C32	0.2569 (2)	−0.0025 (3)	0.33125 (6)	0.0356 (6)	
H32	0.262212	−0.003532	0.308906	0.043*	
C33	0.1620 (2)	0.0739 (3)	0.34423 (6)	0.0321 (5)	
H33	0.101780	0.122515	0.330806	0.039*	
C34	0.1546 (2)	0.0799 (3)	0.37690 (6)	0.0271 (5)	
C35	0.31273 (18)	0.0743 (3)	0.45111 (6)	0.0249 (5)	
C36	0.41921 (19)	0.1640 (3)	0.44030 (6)	0.0289 (5)	
H36A	0.409477	0.190518	0.417614	0.043*	
H36B	0.485327	0.083918	0.444132	0.043*	
H36C	0.432381	0.276140	0.452092	0.043*	
C37	0.28864 (18)	0.0540 (3)	0.48381 (5)	0.0241 (5)	
C38	0.1816 (2)	−0.0267 (3)	0.49249 (6)	0.0278 (5)	
C39	0.1615 (2)	−0.0380 (3)	0.52530 (6)	0.0296 (5)	
H39	0.093225	−0.094292	0.531661	0.036*	
C40	0.2361 (2)	0.0283 (3)	0.54747 (6)	0.0319 (5)	
C41	0.36795 (19)	0.1218 (3)	0.50804 (5)	0.0255 (5)	
C42	0.2234 (3)	0.0326 (4)	0.58228 (7)	0.0551 (8)	
H42A	0.216740	0.157455	0.589305	0.083*	
H42B	0.290943	−0.023040	0.593099	0.083*	
H42C	0.154117	−0.033987	0.587386	0.083*	

### Atomic displacement parameters (Å^2^)

**Table:**

	*U*^11^	*U*^22^	*U*^33^	*U*^12^	*U*^13^	*U*^23^
O1	0.0348 (9)	0.0391 (10)	0.0249 (9)	−0.0058 (7)	−0.0089 (7)	−0.0014 (7)
O2	0.0313 (9)	0.0448 (10)	0.0255 (9)	0.0051 (7)	−0.0051 (7)	0.0043 (7)
O3	0.0324 (9)	0.0441 (10)	0.0293 (10)	0.0025 (8)	−0.0107 (7)	0.0057 (8)
O4	0.0406 (10)	0.0476 (11)	0.0258 (9)	−0.0066 (8)	−0.0063 (8)	0.0060 (8)
N1	0.0270 (10)	0.0296 (10)	0.0227 (10)	0.0011 (8)	−0.0041 (8)	−0.0006 (8)
C1	0.0312 (12)	0.0259 (12)	0.0218 (12)	0.0054 (9)	−0.0043 (9)	−0.0002 (9)
C2	0.0303 (12)	0.0316 (13)	0.0270 (13)	0.0023 (9)	−0.0004 (10)	−0.0009 (10)
C3	0.0352 (13)	0.0358 (14)	0.0349 (15)	0.0053 (10)	0.0039 (11)	0.0033 (11)
C4	0.0443 (14)	0.0404 (14)	0.0242 (13)	0.0105 (11)	0.0056 (11)	0.0008 (11)
C5	0.0425 (14)	0.0345 (13)	0.0210 (12)	0.0063 (10)	−0.0051 (11)	−0.0045 (10)
C6	0.0336 (12)	0.0263 (12)	0.0233 (12)	0.0025 (9)	−0.0039 (10)	0.0002 (9)
C7	0.0246 (11)	0.0260 (11)	0.0247 (12)	−0.0041 (9)	−0.0067 (9)	0.0025 (9)
C8	0.0283 (12)	0.0327 (13)	0.0280 (13)	0.0007 (9)	−0.0063 (10)	0.0017 (10)
C9	0.0266 (11)	0.0300 (12)	0.0232 (12)	−0.0029 (9)	−0.0068 (9)	0.0012 (9)
C10	0.0306 (12)	0.0284 (12)	0.0259 (13)	−0.0046 (9)	−0.0057 (10)	0.0024 (10)
C11	0.0266 (11)	0.0381 (13)	0.0224 (12)	−0.0026 (10)	0.0001 (9)	−0.0011 (10)
C12	0.0345 (13)	0.0413 (14)	0.0241 (13)	−0.0081 (11)	−0.0009 (10)	0.0002 (10)
C13	0.0341 (13)	0.0309 (12)	0.0238 (12)	−0.0073 (10)	−0.0056 (10)	0.0016 (10)
C14	0.066 (2)	0.073 (2)	0.0297 (16)	−0.0211 (17)	0.0054 (15)	−0.0052 (15)
O5	0.0311 (9)	0.0378 (9)	0.0255 (9)	−0.0043 (7)	−0.0069 (7)	0.0001 (7)
O6	0.0313 (9)	0.0514 (11)	0.0301 (10)	0.0049 (8)	−0.0010 (8)	0.0032 (8)
O7	0.0354 (10)	0.0425 (10)	0.0328 (10)	0.0009 (8)	−0.0125 (8)	0.0065 (8)
O8	0.0585 (12)	0.0394 (10)	0.0262 (10)	−0.0038 (9)	−0.0102 (9)	0.0012 (8)
N2	0.0243 (9)	0.0356 (11)	0.0206 (10)	−0.0007 (8)	−0.0055 (8)	0.0013 (8)
C15	0.0291 (12)	0.0282 (12)	0.0214 (12)	0.0063 (9)	−0.0036 (9)	0.0008 (9)
C16	0.0260 (11)	0.0308 (13)	0.0320 (14)	0.0050 (9)	−0.0030 (10)	0.0022 (10)
C17	0.0340 (13)	0.0326 (13)	0.0330 (14)	0.0084 (10)	0.0049 (11)	0.0049 (10)
C18	0.0433 (14)	0.0375 (14)	0.0238 (13)	0.0132 (11)	0.0002 (11)	0.0016 (10)
C19	0.0354 (13)	0.0334 (13)	0.0256 (13)	0.0080 (10)	−0.0069 (10)	−0.0025 (10)
C20	0.0270 (11)	0.0291 (12)	0.0248 (12)	0.0050 (9)	−0.0043 (9)	0.0010 (9)
C21	0.0266 (11)	0.0250 (11)	0.0258 (13)	−0.0042 (9)	−0.0071 (10)	0.0018 (9)
C22	0.0307 (12)	0.0352 (13)	0.0295 (13)	0.0046 (10)	−0.0063 (10)	0.0018 (10)
C23	0.0309 (12)	0.0301 (12)	0.0231 (12)	−0.0040 (9)	−0.0057 (10)	0.0009 (9)
C24	0.0361 (13)	0.0328 (13)	0.0265 (13)	−0.0045 (10)	−0.0032 (10)	0.0018 (10)
C25	0.0436 (14)	0.0335 (13)	0.0273 (13)	−0.0027 (11)	0.0070 (11)	−0.0030 (10)
C26	0.0639 (18)	0.0306 (14)	0.0277 (14)	−0.0055 (12)	0.0029 (13)	−0.0008 (11)
C27	0.0395 (14)	0.0300 (13)	0.0256 (13)	−0.0072 (10)	−0.0092 (10)	0.0016 (10)
C28	0.125 (3)	0.053 (2)	0.0296 (17)	0.000 (2)	0.013 (2)	0.0006 (14)
O9	0.0282 (8)	0.0377 (9)	0.0247 (9)	0.0051 (7)	−0.0046 (7)	0.0013 (7)
O10	0.0250 (8)	0.0416 (10)	0.0293 (9)	−0.0021 (7)	−0.0033 (7)	−0.0011 (7)
O11	0.0274 (8)	0.0390 (9)	0.0303 (9)	−0.0009 (7)	−0.0085 (7)	−0.0040 (7)
O12	0.0383 (9)	0.0399 (10)	0.0253 (9)	0.0056 (7)	−0.0055 (7)	−0.0012 (7)
N3	0.0243 (9)	0.0305 (10)	0.0235 (10)	0.0006 (8)	−0.0028 (8)	0.0004 (8)
C29	0.0285 (11)	0.0270 (12)	0.0220 (12)	−0.0020 (9)	−0.0033 (9)	0.0005 (9)
C30	0.0308 (12)	0.0292 (12)	0.0308 (14)	−0.0035 (9)	−0.0014 (10)	0.0001 (10)
C31	0.0349 (13)	0.0326 (13)	0.0354 (15)	−0.0036 (10)	0.0067 (11)	−0.0028 (11)
C32	0.0462 (15)	0.0381 (14)	0.0227 (13)	−0.0071 (11)	0.0028 (11)	−0.0011 (10)
C33	0.0354 (13)	0.0366 (13)	0.0236 (13)	−0.0038 (10)	−0.0064 (10)	0.0042 (10)
C34	0.0291 (12)	0.0276 (12)	0.0243 (12)	−0.0047 (9)	−0.0024 (10)	0.0005 (9)
C35	0.0231 (11)	0.0253 (11)	0.0257 (12)	0.0045 (8)	−0.0055 (9)	−0.0004 (9)
C36	0.0255 (11)	0.0332 (13)	0.0276 (13)	0.0010 (9)	−0.0031 (10)	−0.0004 (10)
C37	0.0244 (11)	0.0265 (11)	0.0207 (12)	0.0055 (9)	−0.0042 (9)	−0.0018 (9)
C38	0.0286 (12)	0.0261 (12)	0.0281 (13)	0.0044 (9)	−0.0029 (10)	0.0000 (9)
C39	0.0283 (12)	0.0333 (13)	0.0274 (13)	0.0043 (9)	0.0035 (10)	0.0046 (10)
C40	0.0365 (13)	0.0344 (13)	0.0246 (13)	0.0107 (10)	0.0010 (10)	0.0039 (10)
C41	0.0274 (12)	0.0265 (12)	0.0221 (12)	0.0065 (9)	−0.0041 (9)	0.0001 (9)
C42	0.079 (2)	0.0551 (19)	0.0318 (16)	0.0109 (16)	0.0088 (16)	0.0042 (14)

### Geometric parameters (Å, º)

**Table:**

O1—C6	1.361 (3)	C19—C20	1.389 (3)
O1—H1B	0.8700	C19—H19	0.9500
O2—C10	1.253 (3)	C21—C23	1.432 (3)
O3—C13	1.228 (3)	C21—C22	1.497 (3)
O4—C12	1.381 (3)	C22—H22A	0.9800
O4—C13	1.389 (3)	C22—H22B	0.9800
N1—C7	1.326 (3)	C22—H22C	0.9800
N1—C1	1.420 (3)	C23—C27	1.430 (3)
N1—H1A	0.9101	C23—C24	1.445 (3)
C1—C2	1.392 (3)	C24—C25	1.432 (4)
C1—C6	1.397 (3)	C25—C26	1.341 (4)
C2—C3	1.373 (4)	C25—H25	0.9500
C2—H2	0.9500	C26—C28	1.491 (4)
C3—C4	1.393 (4)	C28—H28A	0.9800
C3—H3	0.9500	C28—H28B	0.9800
C4—C5	1.384 (4)	C28—H28C	0.9800
C4—H4	0.9500	O9—C34	1.360 (3)
C5—C6	1.390 (3)	O9—H9B	0.8701
C5—H5	0.9500	O10—C38	1.257 (3)
C7—C9	1.428 (3)	O11—C41	1.225 (3)
C7—C8	1.495 (3)	O12—C40	1.377 (3)
C8—H8A	0.9800	O12—C41	1.400 (3)
C8—H8B	0.9800	N3—C35	1.322 (3)
C8—H8C	0.9800	N3—C29	1.422 (3)
C9—C13	1.435 (3)	N3—H3A	0.9101
C9—C10	1.448 (3)	C29—C30	1.390 (3)
C10—C11	1.430 (3)	C29—C34	1.398 (3)
C11—C12	1.337 (3)	C30—C31	1.382 (4)
C11—H11	0.9500	C30—H30	0.9500
C12—C14	1.487 (4)	C31—C32	1.392 (4)
C14—H14A	0.9800	C31—H31	0.9500
C14—H14B	0.9800	C32—C33	1.382 (4)
C14—H14C	0.9800	C32—H32	0.9500
O5—C20	1.363 (3)	C33—C34	1.389 (3)
O5—H5B	0.8700	C33—H33	0.9500
O6—C24	1.253 (3)	C35—C37	1.433 (3)
O7—C27	1.225 (3)	C35—C36	1.500 (3)
O8—C26	1.381 (4)	C36—H36A	0.9800
O8—C27	1.391 (3)	C36—H36B	0.9800
N2—C21	1.320 (3)	C36—H36C	0.9800
N2—C15	1.424 (3)	C37—C41	1.434 (3)
N2—H2A	0.9101	C37—C38	1.448 (3)
C15—C16	1.390 (3)	C38—C39	1.422 (3)
C15—C20	1.396 (3)	C39—C40	1.338 (4)
C16—C17	1.384 (4)	C39—H39	0.9500
C16—H16	0.9500	C40—C42	1.488 (4)
C17—C18	1.390 (4)	C42—H42A	0.9800
C17—H17	0.9500	C42—H42B	0.9800
C18—C19	1.391 (4)	C42—H42C	0.9800
C18—H18	0.9500		
			
C6—O1—H1B	110.7	C21—C22—H22B	109.5
C12—O4—C13	121.9 (2)	H22A—C22—H22B	109.5
C7—N1—C1	126.7 (2)	C21—C22—H22C	109.5
C7—N1—H1A	111.6	H22A—C22—H22C	109.5
C1—N1—H1A	121.7	H22B—C22—H22C	109.5
C2—C1—C6	120.4 (2)	C27—C23—C21	119.8 (2)
C2—C1—N1	121.4 (2)	C27—C23—C24	119.5 (2)
C6—C1—N1	118.1 (2)	C21—C23—C24	120.6 (2)
C3—C2—C1	120.1 (2)	O6—C24—C25	118.2 (2)
C3—C2—H2	120.0	O6—C24—C23	123.9 (2)
C1—C2—H2	120.0	C25—C24—C23	117.9 (2)
C2—C3—C4	119.7 (2)	C26—C25—C24	120.7 (3)
C2—C3—H3	120.2	C26—C25—H25	119.6
C4—C3—H3	120.2	C24—C25—H25	119.6
C5—C4—C3	120.8 (2)	C25—C26—O8	121.8 (2)
C5—C4—H4	119.6	C25—C26—C28	127.8 (3)
C3—C4—H4	119.6	O8—C26—C28	110.5 (3)
C4—C5—C6	119.8 (2)	O7—C27—O8	113.2 (2)
C4—C5—H5	120.1	O7—C27—C23	128.6 (2)
C6—C5—H5	120.1	O8—C27—C23	118.2 (2)
O1—C6—C5	123.7 (2)	C26—C28—H28A	109.5
O1—C6—C1	117.0 (2)	C26—C28—H28B	109.5
C5—C6—C1	119.3 (2)	H28A—C28—H28B	109.5
N1—C7—C9	117.5 (2)	C26—C28—H28C	109.5
N1—C7—C8	119.1 (2)	H28A—C28—H28C	109.5
C9—C7—C8	123.4 (2)	H28B—C28—H28C	109.5
C7—C8—H8A	109.5	C34—O9—H9B	107.2
C7—C8—H8B	109.5	C40—O12—C41	121.86 (19)
H8A—C8—H8B	109.5	C35—N3—C29	127.1 (2)
C7—C8—H8C	109.5	C35—N3—H3A	111.5
H8A—C8—H8C	109.5	C29—N3—H3A	121.4
H8B—C8—H8C	109.5	C30—C29—C34	120.2 (2)
C7—C9—C13	120.0 (2)	C30—C29—N3	121.2 (2)
C7—C9—C10	120.6 (2)	C34—C29—N3	118.5 (2)
C13—C9—C10	119.3 (2)	C31—C30—C29	120.3 (2)
O2—C10—C11	118.8 (2)	C31—C30—H30	119.9
O2—C10—C9	123.7 (2)	C29—C30—H30	119.9
C11—C10—C9	117.5 (2)	C30—C31—C32	119.4 (2)
C12—C11—C10	121.5 (2)	C30—C31—H31	120.3
C12—C11—H11	119.2	C32—C31—H31	120.3
C10—C11—H11	119.2	C33—C32—C31	120.8 (2)
C11—C12—O4	121.4 (2)	C33—C32—H32	119.6
C11—C12—C14	126.1 (3)	C31—C32—H32	119.6
O4—C12—C14	112.5 (2)	C32—C33—C34	120.1 (2)
O3—C13—O4	114.2 (2)	C32—C33—H33	120.0
O3—C13—C9	127.4 (2)	C34—C33—H33	120.0
O4—C13—C9	118.3 (2)	O9—C34—C33	123.0 (2)
C12—C14—H14A	109.5	O9—C34—C29	117.8 (2)
C12—C14—H14B	109.5	C33—C34—C29	119.2 (2)
H14A—C14—H14B	109.5	N3—C35—C37	117.7 (2)
C12—C14—H14C	109.5	N3—C35—C36	119.1 (2)
H14A—C14—H14C	109.5	C37—C35—C36	123.2 (2)
H14B—C14—H14C	109.5	C35—C36—H36A	109.5
C20—O5—H5B	111.0	C35—C36—H36B	109.5
C26—O8—C27	121.8 (2)	H36A—C36—H36B	109.5
C21—N2—C15	128.2 (2)	C35—C36—H36C	109.5
C21—N2—H2A	110.0	H36A—C36—H36C	109.5
C15—N2—H2A	121.7	H36B—C36—H36C	109.5
C16—C15—C20	120.8 (2)	C35—C37—C41	120.0 (2)
C16—C15—N2	121.3 (2)	C35—C37—C38	120.2 (2)
C20—C15—N2	117.7 (2)	C41—C37—C38	119.7 (2)
C17—C16—C15	120.0 (2)	O10—C38—C39	118.8 (2)
C17—C16—H16	120.0	O10—C38—C37	123.8 (2)
C15—C16—H16	120.0	C39—C38—C37	117.4 (2)
C16—C17—C18	119.1 (2)	C40—C39—C38	121.9 (2)
C16—C17—H17	120.5	C40—C39—H39	119.1
C18—C17—H17	120.5	C38—C39—H39	119.1
C17—C18—C19	121.3 (2)	C39—C40—O12	121.3 (2)
C17—C18—H18	119.3	C39—C40—C42	127.3 (3)
C19—C18—H18	119.3	O12—C40—C42	111.4 (2)
C20—C19—C18	119.5 (2)	O11—C41—O12	114.2 (2)
C20—C19—H19	120.3	O11—C41—C37	128.0 (2)
C18—C19—H19	120.3	O12—C41—C37	117.8 (2)
O5—C20—C19	123.6 (2)	C40—C42—H42A	109.5
O5—C20—C15	117.2 (2)	C40—C42—H42B	109.5
C19—C20—C15	119.2 (2)	H42A—C42—H42B	109.5
N2—C21—C23	117.4 (2)	C40—C42—H42C	109.5
N2—C21—C22	119.3 (2)	H42A—C42—H42C	109.5
C23—C21—C22	123.2 (2)	H42B—C42—H42C	109.5
C21—C22—H22A	109.5		
			
C7—N1—C1—C2	−52.8 (3)	C22—C21—C23—C24	177.1 (2)
C7—N1—C1—C6	131.6 (2)	C27—C23—C24—O6	177.6 (2)
C6—C1—C2—C3	−3.1 (4)	C21—C23—C24—O6	0.2 (4)
N1—C1—C2—C3	−178.6 (2)	C27—C23—C24—C25	−0.3 (3)
C1—C2—C3—C4	1.1 (4)	C21—C23—C24—C25	−177.6 (2)
C2—C3—C4—C5	1.3 (4)	O6—C24—C25—C26	−176.5 (2)
C3—C4—C5—C6	−1.6 (4)	C23—C24—C25—C26	1.5 (4)
C4—C5—C6—O1	179.0 (2)	C24—C25—C26—O8	−2.0 (4)
C4—C5—C6—C1	−0.3 (4)	C24—C25—C26—C28	178.8 (3)
C2—C1—C6—O1	−176.7 (2)	C27—O8—C26—C25	1.4 (4)
N1—C1—C6—O1	−1.1 (3)	C27—O8—C26—C28	−179.3 (2)
C2—C1—C6—C5	2.7 (3)	C26—O8—C27—O7	−180.0 (2)
N1—C1—C6—C5	178.3 (2)	C26—O8—C27—C23	−0.2 (3)
C1—N1—C7—C9	171.8 (2)	C21—C23—C27—O7	−3.3 (4)
C1—N1—C7—C8	−10.5 (3)	C24—C23—C27—O7	179.4 (2)
N1—C7—C9—C13	−177.6 (2)	C21—C23—C27—O8	177.0 (2)
C8—C7—C9—C13	4.8 (3)	C24—C23—C27—O8	−0.3 (3)
N1—C7—C9—C10	3.0 (3)	C35—N3—C29—C30	53.3 (3)
C8—C7—C9—C10	−174.5 (2)	C35—N3—C29—C34	−130.3 (2)
C7—C9—C10—O2	1.9 (4)	C34—C29—C30—C31	3.0 (4)
C13—C9—C10—O2	−177.5 (2)	N3—C29—C30—C31	179.4 (2)
C7—C9—C10—C11	−178.8 (2)	C29—C30—C31—C32	−0.9 (4)
C13—C9—C10—C11	1.9 (3)	C30—C31—C32—C33	−1.4 (4)
O2—C10—C11—C12	177.4 (2)	C31—C32—C33—C34	1.5 (4)
C9—C10—C11—C12	−2.0 (4)	C32—C33—C34—O9	−178.8 (2)
C10—C11—C12—O4	1.9 (4)	C32—C33—C34—C29	0.7 (4)
C10—C11—C12—C14	−177.7 (3)	C30—C29—C34—O9	176.6 (2)
C13—O4—C12—C11	−1.5 (4)	N3—C29—C34—O9	0.2 (3)
C13—O4—C12—C14	178.1 (2)	C30—C29—C34—C33	−2.9 (3)
C12—O4—C13—O3	−179.1 (2)	N3—C29—C34—C33	−179.3 (2)
C12—O4—C13—C9	1.3 (3)	C29—N3—C35—C37	−175.2 (2)
C7—C9—C13—O3	−0.4 (4)	C29—N3—C35—C36	6.7 (3)
C10—C9—C13—O3	178.9 (2)	N3—C35—C37—C41	−177.8 (2)
C7—C9—C13—O4	179.1 (2)	C36—C35—C37—C41	0.3 (3)
C10—C9—C13—O4	−1.6 (3)	N3—C35—C37—C38	−0.9 (3)
C21—N2—C15—C16	54.1 (3)	C36—C35—C37—C38	177.2 (2)
C21—N2—C15—C20	−129.8 (2)	C35—C37—C38—O10	0.6 (3)
C20—C15—C16—C17	2.8 (3)	C41—C37—C38—O10	177.4 (2)
N2—C15—C16—C17	178.7 (2)	C35—C37—C38—C39	−178.2 (2)
C15—C16—C17—C18	0.1 (3)	C41—C37—C38—C39	−1.3 (3)
C16—C17—C18—C19	−2.4 (4)	O10—C38—C39—C40	−176.6 (2)
C17—C18—C19—C20	1.9 (4)	C37—C38—C39—C40	2.2 (3)
C18—C19—C20—O5	−178.1 (2)	C38—C39—C40—O12	−2.3 (4)
C18—C19—C20—C15	0.9 (3)	C38—C39—C40—C42	177.1 (2)
C16—C15—C20—O5	175.9 (2)	C41—O12—C40—C39	1.3 (3)
N2—C15—C20—O5	−0.2 (3)	C41—O12—C40—C42	−178.2 (2)
C16—C15—C20—C19	−3.2 (3)	C40—O12—C41—O11	179.9 (2)
N2—C15—C20—C19	−179.3 (2)	C40—O12—C41—C37	−0.4 (3)
C15—N2—C21—C23	−177.3 (2)	C35—C37—C41—O11	−3.1 (4)
C15—N2—C21—C22	5.3 (4)	C38—C37—C41—O11	−179.9 (2)
N2—C21—C23—C27	−177.5 (2)	C35—C37—C41—O12	177.30 (19)
C22—C21—C23—C27	−0.2 (3)	C38—C37—C41—O12	0.4 (3)
N2—C21—C23—C24	−0.2 (3)		

### Hydrogen-bond geometry (Å, º)

**Table:**

*D*—H···*A*	*D*—H	H···*A*	*D*···*A*	*D*—H···*A*
O1—H1*B*···O7^i^	0.87	1.79	2.662 (2)	177
N1—H1*A*···O2	0.91	1.72	2.538 (3)	148
C8—H8*C*···O8^ii^	0.98	2.48	3.441 (3)	167
C11—H11···O6	0.95	2.57	3.253 (3)	129
O5—H5*B*···O11^iii^	0.87	1.83	2.689 (2)	170
N2—H2*A*···O6	0.91	1.71	2.539 (3)	151
O9—H9*B*···O3^iv^	0.87	1.82	2.691 (2)	179
N3—H3*A*···O10	0.91	1.71	2.532 (3)	148
C33—H33···O3^iv^	0.95	2.53	3.225 (3)	130
C36—H36*B*···O12^v^	0.98	2.56	3.531 (3)	173

Symmetry codes: (i) −*x*+1/2, *y*−1/2, −*z*+1/2; (ii) *x*+1, *y*, *z*; (iii) −*x*+1, −*y*+1, −*z*+1; (iv) *x*−1, *y*, *z*; (v) −*x*+1, −*y*, −*z*+1.
